# Statin effects on atherosclerotic plaques: regression or healing?

**DOI:** 10.1186/s12916-015-0499-9

**Published:** 2015-10-08

**Authors:** Marcio Sommer Bittencourt, Rodrigo Julio Cerci

**Affiliations:** Center for Clinical and Epidemiological Research, University Hospital and São Paulo State Cancer Institute; University of São Paulo School of Medicine, Av. Lineu Prestes, 2565, São Paulo, 05508-000 Brazil; Preventive Medicine Center, Hospital Israelita Albert Einstein, São Paulo, Brazil; Quanta Diagnóstico e Terapia, Curitiba, Brazil

## Abstract

Despite the well-documented improved survival of coronary heart disease with the use of statins, their effects on atherosclerotic plaques are not yet fully understood. While some studies suggest statins may reduce plaque volume, the reduction is small even with the use of high-dose statins. Due to this small change in plaque volume, other effects of statin therapy on plaques have been proposed. A large meta-analysis by Banach et al. explored statin effects on plaque composition detected by intravascular ultrasound (IVUS). We discuss the mechanisms of plaque composition modification demonstrated in their study and its implications on atherosclerotic plaque stabilization.

ᅟ

Please see related article: http://www.biomedcentral.com/1741-7015/13/229.

## Background

Since the landmark publication of the 4S trial, which demonstrated improved survival with the use of statins [1], a series of studies have consistently documented a clear reduction of cardiovascular events with statin treatment in different settings. The benefit is clearly associated with important low-density lipoprotein (LDL) cholesterol reduction achieved by this class of drugs; however, their effects on atherosclerotic plaques have not yet been fully elucidated.

Initial magnetic resonance imaging (MRI) studies of the aorta suggested that statin use might result in atherosclerotic plaque regression as early as 6 months after initiation of therapy [2]. These findings were further corroborated by initial data using intravascular ultrasound (IVUS) evaluation of individuals treated with very high-dose statins [3]. The potential regression or reversion of the atherosclerotic process brought significant excitement in the cardiovascular community. Many understood that changes to the atherosclerotic plaque burden could better summarize the whole statin effect on atherosclerosis beyond LDL cholesterol reduction itself, although such changes in plaque volume were limited to 1 % reduction of the plaque burden. Based on the potential advantages of this strategy some authors even suggested that plaque regression should be considered a new target for therapy [4].

Some of this excitement slowly faded away due to two important pieces of evidence. First, the association between plaque burden and incident cardiovascular events did not prove to be true for other classes of drugs. For example, while niacin effectively reduced the carotid intima-media thickness (IMT), a surrogate marker of atherosclerosis [5], this was not translated in any clinical benefit on a larger trial [6]. In contrast, while ezetimibe failed to reduce the carotid IMT in the same study [5], its use resulted in a significant reduction of cardiovascular events beyond baseline statin treatment [7]. Second, while even lower statin doses result in additional reduction in the rate of cardiovascular events, the regression of plaque volume with statins was limited only to subgroups treated with higher doses and longer duration [8].

### New insights on the effects of statins on atherosclerotic plaques

A new meta-analysis by Banach et al. [9] reinvestigated the effect of statins on atherosclerosis progression and was recently published in *BMC Medicine*. The authors included nine studies with more than 830 individuals in whom virtual histology data was available. This study supported previous findings that higher statin doses result in significant plaque volume reduction, while lower statin doses does not. More interestingly, the pooled virtual histology data demonstrated that this small change in plaque volume is a poor summary of plaque composition change. On the one hand, the fibro-fatty and necrotic core volumes remained unchanged with statin use; and on the other hand, a significant fibrous plaque volume reduction accompanied a significant increase in the dense calcium volume. The data also suggests that there is a direct correlation between statin intensity used in each study and the effects on fibrous plaques and on dense calcium volumes, despite the limited power of subgroup analysis. Another very recent meta-analysis reached similar conclusions using different analytical techniques [10].

An interesting aspect of plaque composition derived from the study by Banach et al. is the role of coronary calcification in the atherosclerotic process and its implications on cardiovascular risk assessment. While some authors propose that calcium formation is part of the healing and stabilizing process of atherosclerosis, other *in vitro* studies indicate that calcium location may better explain the difference in plaque rupture risk [11]. In fact, while clinical studies have firmly documented a direct association between the overall calcification in coronary arteries (measured as either the Agatston score or calcium volume) and cardiovascular events, other studies suggest that the pattern and distribution of calcium in coronary plaques may equally matter. A classical coronary computed tomography (CT) angiography study by Motoyama et al. [12] indicated that small “spotty” calcifications are associated with future plaque ruptures, whereas a sub-analysis of the Multi-Ethnic Study of Atherosclerosis (MESA) study [13] demonstrated that calcium density is inversely associated with events risk. These results, grounded by cellular and molecular data on the mechanisms regulating the pattern of atherosclerotic calcification, suggest that smaller calcium deposition (pro-inflammatory-driven microcalcifications) are associated with plaque rupture and increased cardiovascular risk, while larger, denser calcium structures (anti-inflammatory-driven macrocalcifications) are associated with plaque stabilization and better outcomes, in agreement with the increased dense calcification documented with statin treatment [14].

Collectively, these results indicate that instead of regression, the use of statins can lead to plaque healing and stabilization. The “healed” plaque is only discretely smaller, although it has better structure and is less prone to rupture. Even so, the occurrence of cardiovascular events despite optimized treatment in a significant proportion of patients receiving statins, the so-called “residual risk”, suggests that the healing process is incomplete. In fact, it has been proposed that statins and other preventive measures alter the mechanisms leading to acute coronary syndromes. Instead of plaque rupture, acute coronary syndromes in those “healed” plaques are more likely to occur by erosion. This change is thought to be related to the improved plaque structure associated with the change in its components.

## Conclusions

In light of the evidence from the study by Banach et al., future clinical studies using invasive and non-invasive techniques should move forward, with a deeper evaluation of drug effects on atherosclerotic plaques. Instead of absolute quantification of plaque and its components (for example, plaque volume), we should focus on the identification of plaque characteristics or changes in the distribution of plaque components (for example, “spotty” calcification, positive remodeling), as plaques are much more likely to heal and stabilize than to regress.

## Abbreviations

CT: Computed tomography
IMT: Intima-media thickness
IVUS: Intravascular ultrasound
LDL: Low-density lipoprotein
MESA: Multi-Ethnic Study of Atherosclerosis
MRI: Magnetic resonance imaging
